# Modelling social norms: an integration of the norm-utility approach with beliefs dynamics

**DOI:** 10.1098/rstb.2023.0027

**Published:** 2024-03-11

**Authors:** Sergey Gavrilets, Denis Tverskoi, Angel Sánchez

**Affiliations:** ^1^ Department of Ecology and Evolutionary Biology, University of Tennessee, Knoxville, TN 37996, USA; ^2^ Department of Mathematics, University of Tennessee, Knoxville, TN 37996, USA; ^3^ Center for the Dynamics of Social Complexity, University of Tennessee, Knoxville, TN 37996, USA; ^4^ Grupo Interdisciplinar de Sistemas Complejos, Departamento de Matemáticas Universidad Carlos III de Madrid, Leganés, Madrid 28911, Spain; ^5^ Instituto de Biocomputación y Física de Sistemas Complejos, Universidad de Zaragoza, Zaragoza 50018, Spain

**Keywords:** behaviour, beliefs, mathematical modelling, game theory, social evolution, cultural evolution

## Abstract

We review theoretical approaches for modelling the origin, persistence and change of social norms. The most comprehensive models describe the coevolution of behaviours, personal, descriptive and injunctive norms while considering influences of various authorities and accounting for cognitive processes and between-individual differences. Models show that social norms can improve individual and group well-being. Under some conditions though, deleterious norms can persist in the population through conformity, preference falsification and pluralistic ignorance. Polarization in behaviour and beliefs can be maintained, even when societal advantages of particular behaviours or belief systems over alternatives are clear. Attempts to change social norms can backfire through cognitive processes including cognitive dissonance and psychological reactance. Under some conditions social norms can change rapidly via tipping point dynamics. Norms can be highly susceptible to manipulation, and network structure influences their propagation. Future models should incorporate network structure more thoroughly, explicitly study online norms, consider cultural variations and be applied to real-world processes.

This article is part of the theme issue ‘Social norm change: drivers and consequences’.

## 1. Background

In social sciences, most definitions of social norms involve beliefs about what others do and about what they should or should not do. The former are called descriptive norms [1], empirical expectations [2] or folkways (emerging out of routines, such as waiting in line). The latter are termed injunctive norms [1], normative expectations [2], mores (specifying what is moral or unethical), taboos (prohibition of behaviours so strict it results in disgust), prescriptive norms (encouraging positive behaviour), and proscriptive norms (discouraging negative behaviour) [3]. Such norms exist because of the collective belief in their existence, something akin to self-fulfilling prophecies [4]. Norms vary among families, cultural, ethnic or religious groups, regions and countries, and are influenced by exposure to different situations, leading to different degrees of adherence often described in terms of societal tightness–looseness [5–8]. Specifically, ‘tight’ cultures display strong norms, low tolerance for deviance, resistance to innovations and uniform social conduct, while ‘loose’ cultures demonstrate more relaxed norms, are more tolerant, and exhibit more diverse conducts. Importantly, people can incorrectly perceive others’ beliefs, leading to pluralistic ignorance: people may believe their private thoughts and feelings differ from those of others when in fact they are not [9–11]. While the two types of norms mentioned above focus on beliefs about others’ actions and beliefs, personal norms (normative beliefs) describe what individuals believe they themselves should do. Personal norms can be shaped by an individual’s moral values, often stemming from considerations about the welfare of others [2,12,13], or from their sense of what actions and beliefs are most appropriate [14,15]. These norms can also evolve from internalized social norms [16]. Here, we adopt a broad interpretation of personal norms, acknowledging that they can change over time. Independently of all these details, the ultimate factors explaining the origin, maintenance and diversity of norms are human susceptibility to social influence [17], pay-off differences between behaviours in different environments, and stochasticity involved in the appearance, spread and disappearance of behaviours in populations.

### (a) Why people follow the norms

There are multiple reasons for people to follow social norms [2,18–22]. Social norms enable individuals to anticipate others’ behaviours, thus leading to smoother social interactions. In uncertain situations, people infer latent norms via observation (when in Rome, do as the Romans do), a self-reinforcing process perpetuating these norms. Various factors such as mimicry, desire for approval and group identity contribute to norm adherence [23–25]. Individuals may also conform with others’ perceived beliefs owing to perceptual and behavioural constraints or to avoid punishment of norm violators [2,19,21,26–29]. Norm internalization, where norms are adopted as personal beliefs and values, also enhances adherence [30–35]. Violation of these internalized norms can cause psychological discomfort, even when associated with material benefits [36]. Norm internalization can reduce costs related to information processing and decision-making [35], and help ensure cooperation [33,35]. While the inclination to follow norms is partly innate, specific norms are culturally influenced [37,38]. However, personal norms may be disregarded under conditions like high-compliance costs. Overall, following social norms is a multifaceted process influenced by individual cognition, group dynamics and broader societal factors.

### (b) How norms change

New norms can emerge in younger generations, driven by a desire for a distinct social identity or competition for resources with older generations [39–44]. Changes in norms can also be triggered by fresh information about costs, benefits or others’ behaviours and beliefs, and by alignment with authoritative or influential individuals. Normative beliefs can be recalibrated by correcting misperceptions about group behaviour and approval. Structural, ecological, historical, economic changes or specific policies that incentivize or regulate behaviours can impact norms and normative systems [45]. Education campaigns and communications by cultural or institutional actors can significantly influence norm changes [46–48]. Social norms can be changed by relatively small groups. As Margaret Mead said, ‘never doubt that a small group of thoughtful, committed citizens can change the world,’ reflecting the potential impact of trendsetters [46] and committed minorities [47,48] on norm evolution. Conversely, norms may persist despite environmental shifts, leading to a cultural mismatch [49].

Below, we discuss the forces and factors essential for modelling norm dynamics realistically. Next, we evaluate existing approaches based on how they incorporate these factors. We identify an emerging integrative approach optimal for modelling norm dynamics and review related work. We conclude by outlining general norm dynamics patterns identified by mathematical models.

## 2. Perspectives on modelling social norms

### (a) Forces and factors to account for in models of norm dynamics

There are several crucial factors that must be accounted for in any realistic theory attempting to describe and predict norm dynamics.

#### (i) Decision-making and beliefs

Human beliefs are crucial in decision-making [50,51] as reflected in what is known in social psychology as the ‘Thomas’ theorem’: ‘If men define situations as real, they are real in their consequences’ [52, pp. 571–572]. Regarding social norms, there are four elements of decision-making where human beliefs are very important. Firstly, empirical expectations influence our decision-making by affecting our anticipated material pay-offs. Secondly, our decisions are also influenced by the psychological well-being derived from conformity, where our behaviour is aligned with perceived norms leading to feelings of belonging and acceptance. Therefore, we are more likely to engage in a behaviour expected in our group even if it contradicts our personal beliefs or interests. Thirdly, normative expectations can significantly influence decision-making: we avoid actions we think will be disapproved to maintain our social standing. Conversely, we may behave in a way we believe will earn approval, even at personal cost. Lastly, personal norms impact our behaviour because they align with our values and self-image. Following them reinforces our self-concept as moral and good individuals, improving our psychological well-being.

#### (ii) Beliefs and attitudes changes

Changes in social norms occur simultaneously with changes in our beliefs about what others do, what others think and what is right or wrong in different situations [53–59]. Some changes occur gradually over generations, such as the norm regarding gender roles in many societies [60]. Other norms can change relatively quickly [61]. Norm change velocity can also be influenced by the level of consensus about a norm and the connectivity in a society or group [62]. Sometimes, the formation of our beliefs is not driven by conscious reasoning but by subconscious anticipation of their potential effects on others. These others can either reward or chastise us—occasionally promoting baseless beliefs, while sometimes penalizing justified ones [29,63]. Thus, integrating belief dynamics into theories of social norms is crucial.

#### (iii) Cognitive and psychological processes

Various psychological and cognitive processes influence decision-making and belief dynamics [64]. Cognitive dissonance, the mental discomfort experienced when holding contradictory beliefs, values or attitudes, can be lessened through behaviour changes, new beliefs or selective memory [65–69]. Social projection, where individuals attribute their own thoughts and feelings to others [70–72], equips individuals with a ‘theory of mind,’ the ability to attribute mental states to oneself and others [73–76]. Another important process is psychological reactance, where individuals resist threats to their freedom, leading to oppositional behaviour or belief reinforcement [77–79]. Emotions also influence decision-making: fear promotes avoidance and conformity, and happiness drives behaviours with immediate rewards [80–84]. Emotions can both stem from and contribute to cognitive dissonance, and assist in understanding others’ mental states.

#### (iv) Between-individual differences

Unique personality traits, cognitive styles, emotional reactions and social experiences can result in between-individual differences crucial in decision-making and belief dynamics. For instance, social identity theory shows that group identification can influence decisions [23,85], while cognitive dissonance theory highlights different strategies for resolving conflicting beliefs [65]. The theory of mind depends on diverse abilities to understand others’ perspectives [73], and social projection underlines individual tendencies to assume shared beliefs, affecting interpretations of social norms [72]. Variations in conformity, anticonformity [86] and psychological reactance [77] affect behaviour in the context of social norms. Neglecting these differences leads to inaccurate predictions of behaviour and ineffective behaviour promotion. Cultural differences also play a significant role [87].

### (b) Theoretical approaches for modelling social norms

There is a very large number of different theories of behavioural change [88] many of which have been studied using mathematical models. Here, we evaluate several approaches most fitting for modelling social norms dynamics in light of their ability to capture the factors discussed above.

### (c) Classical and evolutionary game theory models

Classical non-cooperative game theory relies on utility maximization under perfect rationality of players [89]. By contrast, evolutionary game theory considers bounded rationality through processes like myopic best responses or imitation [90]. Social norms are often seen as equilibria in this context [39,45,91,92]. According to North [91, p. 821], a norm is ‘an established and self-reinforcing pattern of behaviour: everyone wants to play their part given the expectation that everyone else will continue to play theirs. It is, in short, an equilibrium of a game.’ Social norms emerge from interactions impacting individual pay-offs and are reinforced by reduced pay-offs for deviating behaviours, such as miscoordination costs or punishment by peers or institutions [39,90]. In evolutionary game theory, norms can undergo abrupt shifts (tipping) rather than gradual changes. Multiple equilibria are common, resulting in local populations conforming to different norms, maintaining global diversity [39]. Both classical and evolutionary game theories offer valuable frameworks for understanding how pay-off structures can influence behaviour across various scenarios. However, these theories often overlook normative considerations and psychological factors. Evolutionary game theory focuses instead on pre-programmed behavioural responses/strategies like cooperation, defection or punishment of defectors. Individuals in these models are typically assumed to either execute specific actions or imitate those with higher pay-offs, subject to occasional errors. While these approaches excel at capturing descriptive norms, they are less adept at addressing injunctive norms although some models that include punishment mechanisms for free-riders [32,93,94] can be seen as touching on injunctive norms as well.

### (d) Psychological game theory models

Psychological game theory integrates beliefs, emotions and cognitive biases, improving our understanding of human behaviour in strategic situations by recognizing imperfect information and deviations from strict rationality [95–101]. Psychological game theory aims to account for the fact that what you believe others will do or think can actually make you happier or unhappier. For example, a player may experience guilt when he believes that the pay-off of his partner is lower than what the partner expected [101]. These beliefs can then influence the player’s decision-making. Psychological game theory can indirectly model norms by incorporating psychological factors that can be influenced by them, capturing how social norms shape individual expectations about others and how guilt resulting from norm violations affects behaviour. However, existing models focus on anticipated behaviour rather than on normative expectations, making injunctive social norms challenging to model [101–103].

### (e) Social influence models

Social influence mechanisms, such as imitation and conformity with peers, authority figures or high-status individuals can lead to convergence on shared behaviours, even without precise information about costs and benefits [104,105]. Convergence can lead to a consensus or to polarized states where multiple norms coexist within a population [48,104–108]. Norm transmission occurs through imitation and copying within the same generation or across generations. Recent research focuses on how social network structures impact norm dynamics [109–111]. Persistence, tipping, local convergence and global diversity, observed in evolutionary game theory, are present in social influence models. However, they often oversimplify by neglecting strategic behaviour and norm-adherence costs and benefits.

### (f) Norm-utility models

Norm-utility models, a term not widely used (but see [103]), incorporate adherence to or deviation from social norms into individuals’ utility functions [2,103,112–114]. Thus, people’s decisions are not purely based on material considerations, but also on perceptions of what is appropriate or acceptable in a social group. These models usually represent social norms as rules or expectations about appropriate behaviour whose violation leads to a decrease in utility. Thus, behaviour that appears irrational in terms of material pay-offs becomes rational when the utility from norm adherence is considered. They are particularly useful for analysing social dilemmas, cooperation and phenomena where social norms are relevant. However, norm-utility models usually do not consider the changing nature of personal norms or normative expectations.

### (g) The role of beliefs and between-individual differences in models of norm dynamics

The above approaches differ in the role of individual beliefs. Evolutionary game theory embodies beliefs based on expectations about others’ behaviour, and individuals adopt successful or expected-to-be-successful strategies without having explicit beliefs. In psychological game theory, individuals have beliefs about other players’ strategies, intentions and mental states, influencing their decisions and responses. In social influence models, beliefs refer to individuals’ opinions or attitudes influenced by others and are updated based on received information, leading to collective behaviours like consensus or polarization. In norm-utility models, individuals’ beliefs about normative behaviours shape their utility from different actions, so changes in beliefs about norms can drive changes in norm adherence. Regarding between-individual differences, game-theoretic models often neglect them except for strategies, while social influence models ignore them except for opinions and positions in the social network. By contrast, between-individual differences are a crucial component of many norm-utility models.

**Figure 1.**
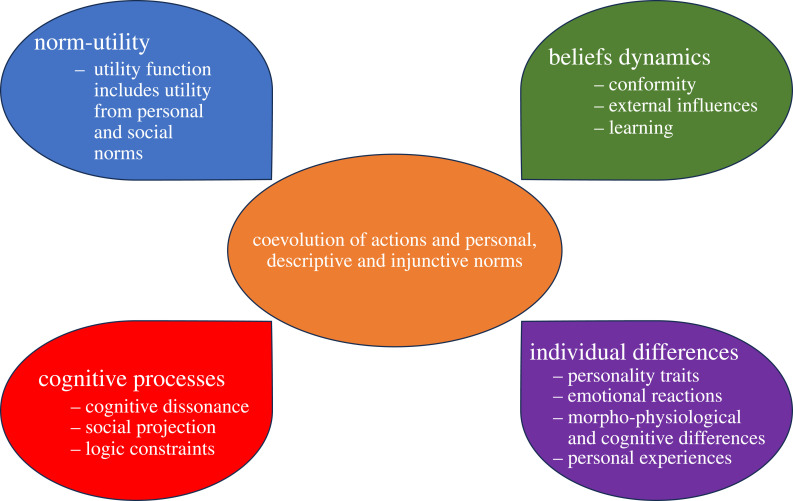
An integrative approach to modelling social norms. (Online version in colour.)

Importantly, as we discussed above, beliefs coevolve with actions. Therefore, adequately modelling social norms requires considering jointly the dynamics of actions, attitudes (personal norms) and beliefs about others while accounting for cognitive processes and between-individual differences (figure 1). This can be achieved by integrating norm-utility approaches with social influence models, as we show in the next section.

## 3. Some specific models of norm dynamics

In this section, we look into the details of specific norm-utility models in some of which decision-making coevolves with norms. Along our discussion, we might occasionally simplify these models, omitting certain components for clarity. We will also modify and streamline notations for ease of understanding and comparison. Even if the original models did not explicitly centre on social norms, we aim to interpret their implications and conclusions in that context. In all models we consider, individuals choose the action maximizing the utility function. If belief dynamics are considered, they are usually described by simple linear equations that extend the classical DeGroot model of opinion change [107,115]. These extended models account for additional factors that influence individual beliefs (e.g. cognitive dissonance, social projection or authority’s messaging), beyond just the opinions of peers. In a few cases, the changes in both actions and beliefs are found by a joint maximization of the utility function with respect to variables describing beliefs dynamics. We will organize the models based on the variables that undergo dynamic changes—whether these are merely actions or also personal norms and beliefs about others. We will discuss modelling assumptions about factors controlling decision-making (via utility function) and belief dynamics as well as main conclusions. For the purpose of our discussion, we will adopt the following notations: *x* for action (behaviour), *y* for personal norm (intrinsic preference or attitude), $\tilde{x}$ for first-order belief (empirical expectation), $\tilde{y}$ for second-order belief (normative expectation), $\bar{x}$ for (average) observed behaviour of peers, *π* for material pay-offs, *u* for the utility function. Coefficients *A*_0_, *A*_1_, *A*_2_, *A*_3_ and *A*_4_ will capture the effects of material factors, personal, descriptive, injunctive norms and authorities, respectively, on individual decision-making. We will use this notation throughout the paper irrespective of the ones used in the original papers so all models can be meaningfully compared with each other.

The relationships between our main variables $x,y,\tilde{x},\tilde{y}$ in social dilemmas have been extensively studied through behavioural experiments and heuristic regression models. For example, Fischbacher & Gächter [116] used multiple-round public goods experiments to study the effects of empirical expectations on actions and of observed behaviour on normative expectations (see also [117–119]). Bicchieri & Xiao [120] contrasted the effects of empirical and normative expectations in the dictator game. Other types of social interactions have also been studied [114,121–124]. Decisions to cooperate strongly depend on whether others are expected to do so [28]. Informing subjects about both peers’ actions and beliefs lead to synergistic effects [125]. Empirical and normative expectations can interact with personal norms [126–128]. The effort of authorities can change perceived norms [129]. People can strategically distort their beliefs, including those about norms, to justify self-serving behaviour [130–132]. All these findings highlight further the necessity of explicitly modelling the coevolution of beliefs and decision-making to understand behaviour in social dilemmas.

### (a) Early norm-utility models in economics

Early norm-utility models had a significant impact on subsequent research in economics. The pioneering paper by Akerlof [112] modelled complex interactions between labourers and capitalists incorporating consumption, reputation and action-belief alignment into the utility function. Individuals adhered (*x* = 1) or not (*x* = 0) to a norm, believing (*y* = 1) or not (*y* = 0) in it. A reputational loss proportional to norm supporter frequency ($\bar{y}$) occurs upon norm-breaking. These assumptions lead to the utility function:

3.1 $$u(x)=\underset{\mathrm{material}\;\mathrm{pay-off}}{\underbrace{A_{0}\pi (x)}}-\underset{\mathrm{cognitive}\;\mathrm{dissonance}}{\underbrace{A_{1}(1-x)y}}-\underset{\mathrm{reputational}\;\mathrm{loss}}{\underbrace{A_{2}(1-x)\bar{y},}}$$

where *π*(*x*) is the material pay-off resulting from action *x* and constant parameters *A*_*i*_ measure the relative weights of the corresponding factors. Akerlof focused on norms that decrease individual pay-offs (*π*(1) < *π*(0)). The model predicts heterogeneity in both actions and beliefs with disadvantageous norms persisting because breaking them results in reputation loss. The model also predicts that individuals may adhere to norms even if they personally disagree with them (preference falsification [133]).

Follow-up papers applied this approach to several cases. A model on workplace safety beliefs [134] incorporates fear-induced mental costs into economic modelling, providing insights into the spread of innovations, advertisement influences, social security necessity and aspects of crime. A crime model [135] showed that cognitive dissonance can influence individuals to choose criminal activities under harsh penalties but dissuades them when penalties are lenient. Akerlof & Kranton [136,137] modelled situations where individuals optimize utility by selecting effort levels (*x*) and identities (*y*), looking at students who exert effort in academic pursuits and classify themselves into ‘leading crowd,’ ‘nerds’ and ‘burnouts,’ each with distinct behavioural norms.

Rabin [138] models the impact of cognitive dissonance on immoral behaviour, such as wearing fur, allowing continuous variation in actions (*x*) and moral beliefs (*y*). Individual pay-offs *π*(*x*) increase with engagement level *x*, but excessive levels may be morally unacceptable. If *x* > *y*, cognitive dissonance induced a utility loss *d*(*x* − *y*). Maintaining morally wrong beliefs (*y*) also led to a psychic cost (*c*(*y*)). With these assumptions we have:

3.2 $$u(x,y)=\underset{\mathrm{material}\;\mathrm{pay-off}}{\underbrace{A_{0}\pi (x)}}-\underset{\mathrm{cognitive}\;\mathrm{dissonance}}{\underbrace{A_{1}d(x-y)}}-\underset{\mathrm{cost}\;\mathrm{of}\;\mathrm{holding}\;\mathrm{belief}}{\underbrace{A_{2}\;c(y).}}$$

Maximizing the utility function *u* by considering simultaneously actions (*x*) and beliefs (*y*), Rabin showed that amplifying aversion to immorality (raising costs *c*) can paradoxically increase immoral behaviours owing to cognitive dissonance, where individuals attempt to rationalize immoral behaviours as morally acceptable. When individuals’ beliefs influence one another, heightened immorality discomfort can unwittingly encourage collective rationalization of questionable activities, escalating their prevalence. If individuals are primarily influenced by observable behaviours of others rather than by expressed beliefs, increasing the perceived cost of immorality would lead to a decline in immoral activities.

Bernheim [139] modelled individuals who receive material benefits and utility from the prestige granted to them by others, with actions represented by a continuous variable *x*. Individuals differ in the type *θ* specifying the action that produces the highest material pay-off. Social interactions are implicit rather than explicit, with the assumption of a universally recognized most prestigious type set at *θ* = 1. Then, we have:

3.3 $$u(x)=\underset{\mathrm{material}\;\mathrm{pay-off}}{\underbrace{-A_{0}{\left(x-\theta \right)}^{2}}}-\underset{\;\mathrm{prestige}\;\mathrm{loss}}{\underbrace{A_{2}{\left(1-x\right)}^{2}.}}$$

The model reveals that when societal status outweighs individual preferences (large *A*_2_), many individuals conform to a uniform behavioural standard, disregarding their own preferences. However, groups with significant variation in individual preferences (*θ*) can resist conformity. The model clarifies why some activities follow behavioural standards while others do not, provides insights into norm evolution owing to preference shifts, and can explain both enduring customs and transient fads. Bénabou & Tirole [140] explored a similar model where the prestige of an action increased with its frequency. They identified conditions for two equilibrium states, each represented by the unanimous selection of one action or the other by all individuals involved. A later paper by the same authors [141] allowed for variation between individuals in intrinsic motivation *y* to perform a particular action. They used the model to explore the effects of norm-based interventions (such as making descriptive and injunctive norms more salient), aiming to increase the group’s welfare.

### (b) The Rashevsky model

Next we describe two classical models which initially were formulated without a consideration of utility function but nevertheless can be viewed as examples of the norm-utility approach. By contrast to the models discussed above, these two models are dynamic, directly capture conformity with peer behaviour, and explicitly account for the difference between individuals in characteristics controlling decision-making. The model developed by Rashevsky [106] was the very first attempt to model the effects of social influences on behaviour. (Nicolas Rashevsky is also viewed as the founder of mathematical biology [142] and cliodynamics [143].) Consider a population of *N* individuals who can take two actions: *x* = 0 and *x* = 1. The probability *P* of taking action 1 is monotonically increasing with the latent ‘position’ of the individual with regards to these two actions, written as a sum *y* + *z*, where *y* is a constant personal attitude which may depend on expected material or immaterial values associated with the actions. The term *z* is the net effect of social influence, assumed to be equal for all individuals. Building on a model of neural discrimination between stimuli [144], Rashevsky [106] described the dynamics of social influence *z* by a differential equation:

3.4 $$\frac{dz}{dt}=\underset{\mathrm{effect}\;\mathrm{of}\;\mathrm{conformity}}{\underbrace{\alpha N[2p(z)-1]}}-\underset{\mathrm{decay}\;\mathrm{of}\;\mathrm{social}\;\mathrm{influence}}{\underbrace{\beta z,}}$$

where *p*(*z*) is the frequencies of behaviour 1 in the population. If behaviour 1 is more common (i.e. *p*(*z*) > 0.5), the first term describes an increase in *z*, otherwise it describes a decrease in *z*. The second term describes the decay of social influence to zero. Constant parameters *α* and *β* scale the corresponding rates of change in social influence. The model is completed by specifying the density function of the distribution *f*(*y*) of personal attitudes *y* in the population and the function *P* converting *y* + *z* into the probability of choosing action *x* = 1. Given these two functions, p(z)=∫P(y+z)f (y) dy.

Rashevsky [106] demonstrated that *z* evolves towards an equilibrium, but also that there can be multiple equilibria, hence the final outcome may depend on initial conditions. The population can become ‘stuck’ in a state where a non-preferred behaviour or norm is maintained. Rashevsky’s findings underscore the significance of heterogeneity in attitude *y* as characterized by function *f*(*y*). Small parameter changes can induce tipping point dynamics and sudden shifts in population behaviour. Recent studies have used this model to examine interactions between identity groups and the effects of identity salience and propaganda on group behaviour [145–147].

### (c) Granovetter-type models

The model formulated by Granovetter [48] is a generalization of models of spatial segregation developed by Schelling [148,149]. The beauty of Granovetter’s formulation is in its simplicity. The model was introduced within the context of riots or social protests which each individual can join (*x* = 1) or not (*x* = 0) but we can also think about it in terms of other behaviours and norms. Each individual is characterized by a threshold *d* such that if the frequency $\bar{x}$ of others choosing action 1 is larger than *d*, the individual does the same. The actual value of *d* may depend on the perceived costs and benefits of possible actions, on personality, etc. The cumulative distribution of thresholds *F* in the population is assumed to be constant in time. Then if the current frequency of people choosing action 1 is ${\bar{x}}_{t}$, then for the proportion $F({\bar{x}}_{t})$ of the population ${\bar{x}}_{t}$ is larger than their thresholds, so they will choose action 1 as well. This immediately leads to a recurrence equation describing the dynamics of $\bar{x}$:

$${\bar{x}}_{t+1}=F({\bar{x}}_{t}).$$

It can be shown that, as time increases, ${\bar{x}}_{t}$ converges to an equilibrium. There can be several equilibria ${\bar{x}}^{*}$, which are given by solutions of the algebraic equation ${\bar{x}}^{*}=F({\bar{x}}^{*})$. The Granovetter model can also be formulated in continuous time [150].

**Figure 2.**
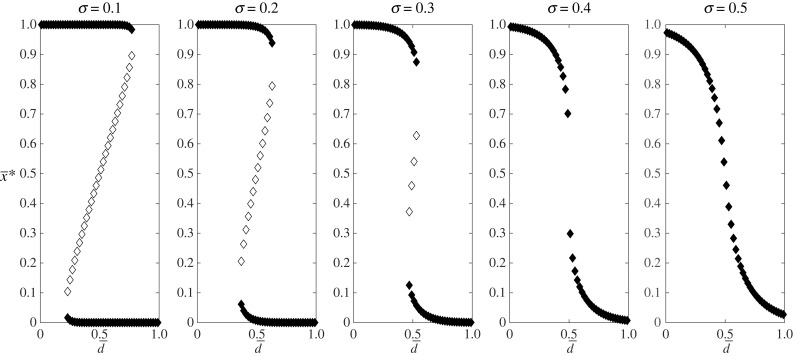
Equilibrium values of frequency ${\bar{x}}^{*}$ for the Granovetter model (3.5) assuming a truncated normal distribution of thresholds with mean $\bar{d}$ and variance *σ*^2^. Stable equilibria are shown with solid circles; unstable equilibria are shown with open circles.

To analyse the model in more detail we must specify the cumulative distribution function *F*(*d*). Figure 2, shows the equilibria when the distribution of thresholds is truncated normal with mean $\bar{d}$ and variance *σ*^2^. When *σ* is small while $\bar{d}$ is intermediate, there are two stable equilibria (close to $\bar{x}=0$ and $\bar{x}=1$) and an unstable equilibrium with intermediate $\bar{x}$. In this case, there is possibility for a tipping point dynamic when a small change in parameters can cause a dramatic change in the equilibrium frequency of behaviour. For example, in the left most figure increasing $\bar{d}$ beyond approximately 0.77 will cause $\bar{x}$ to drop from about 1 to about 0 while decreasing $\bar{d}$ beyond approximately 0.23 will cause $\bar{x}$ to increase from about 0 to about 1.

Yin [151] contrasted the cases where *F* is unimodal or bimodal, with equal or unequal peak values, to assess the effectiveness of interventions in promoting or suppressing mass protests. Efferson *et al.* [152] considered the effects of changing the distribution from a unimodal to bimodal (e.g. by educating a certain proportion of the population) to eliminate harmful social norms, such as female genital cutting.

Neither Rashevsky nor Granovetter had much to say about the nature of attitudes/thresholds *y* which were rather abstract in their models, but they can be linked to psychological factors and forces involved in decision-making. For example, in the model introduced by Kuran [153] individuals suffer moral integrity costs based on the discrepancy between their private attitude *y* and the action *x* taken, but receive reputational benefits proportional to the frequency of those exhibiting the same behaviour. Then the utility function becomes:

3.6 $$u(x)=-\underset{\mathrm{moral}\;\mathrm{integrity}\;\mathrm{cost}}{\underbrace{A_{1}|x-y|}}-\underset{\mathrm{reputational}\;\mathrm{benefit}}{\underbrace{A_{3}|x-\bar{x}|.\;}}$$

In this model, the threshold value of $\bar{x}$ at which the utility of action (*x* = 1) becomes larger than that of non-action (*x* = 0) is *d* = ((*A*_1_+*A*_2_)/2*A*_2_) − (*A*_1_/*A*_2_)*y*, so that a larger attitude *y* means a smaller threshold *d*. Now one can use equation (3.5) to describe the dynamics of the frequency ${\bar{x}}_{t}$ of people participating in mass protest. All conclusions from the original Granovetter model apply here.

Centola *et al.* [154] studied why people publicly enforce a norm that they secretly wish would go away. In their model, people can privately support (*y* = 1) or oppose (*y* = −1) the norm, comply (*x* = 1) or not (*x* = −1) with the norm, and punish (*z* = 1) norm violators, punish norm-followers (*z* = −1) or do not punish anybody (*z* = 0). Mean values $\bar{x}$ and $\bar{z}$ measure the extent of compliance and punishment in the population. At the first step of each round, each individual decides whether to comply or not with the norm by choosing an action *x* maximizing

3.7a $$u(x)=\underset{\mathrm{cognitive}\;\mathrm{dissonance}}{\underbrace{A_{1}xy}}+\underset{\mathrm{social}\;\mathrm{pressure}}{\underbrace{A_{2}x\bar{z}.}}$$

The first term is maximized if action *x* and belief *y* match, while the second is maximized when the action complies with the prevailing punishment in the population (i.e. the sign of *x* matches that of $\bar{z}$).

At the second step, individuals who acted according to their beliefs at the first stage (i.e. those with *x* = *y*) can punish people with deviating behaviour if the need for enforcement (measured by $w=(1-y\bar{x})/2$) is sufficiently large. Those who acted against their beliefs because of social pressure, can follow with a ‘false enforcement’, that is, punish people whose behaviour they privately approve if social pressure is strong enough. These assumptions lead to two separate utility functions:

3.7b $$u(z|x=y)=-\underset{\mathrm{cost}\;\mathrm{of}\;\mathrm{punishing}}{\underbrace{cyz}}+\underset{\;\mathrm{personal}\;\mathrm{norm}}{\underbrace{A_{1}wyz,}}$$

and

3.7c $$u(z|x=-y)=-\underset{\mathrm{cost}\;\mathrm{of}\;\mathrm{punishing}}{\underbrace{(A_{1}+c)yz}}+\underset{\mathrm{social}\;\mathrm{pressure}}{\underbrace{\bar{z}z,}}$$

where *c* measures the cost of punishing others. Strong conviction (larger *A*_1_) promotes true enforcement (equation (3.7*b*)) and inhibits false enforcement (equation (3.7*c*)). Centola *et al.* [154] numerically studied this model on social networks, showing that when interactions between small neighbourhoods are limited, a small group can ignite cascades leading to almost universal norm adherence and enforcement. Converting false enforcers into true believers does not stabilize high-compliance equilibrium, but instead can trigger its collapse. Certain network features known for promoting the spread of information, innovations, rumours and diseases [155], hinder cascades of false enforcement.

Gavrilets [156] examined a model where individuals can adopt traditional or new behaviours. The traditional behaviour persists owing to its normative status, despite costs. Individuals gain approval or face disapproval based on behavioural alignment with others. Norm-followers have the option to punish norm-violators at a personal cost. The model’s dynamics are defined by two Granovetter-type equations for the frequencies of norm-followers and punishers. The model shows that unpopular norms can persist owing to preference falsification, emphasizing the impact of parameters and initial conditions. Changes in the distribution of personal norms can significantly alter norm adherence frequency. Minor parameter adjustments can cause significant societal shifts, and behaviour modifications can be achieved by altering costs, normative values, societal expectations and strategic information dissemination. Gavrilets [156] discusses policy implications in abolishing norms such as footbinding and female genital cutting, reducing college students’ drinking and promoting pro-environmental behaviours.

McCullen *et al.* [157] proposed a Granovetter-type model with thresholds being a weighted combination of behaviour frequencies across the entire system and the local neighbourhood. Their findings emphasize two crucial elements influencing the dynamics: the number of connections a node has with its neighbours, and the network’s transitivity or clustering, that correlates with the neighbourhoods of interconnected individuals.

### (d) Other models of the dynamics of descriptive norms

Norm-utility models in which personal norms/attitudes do not change, predict changes in the average behaviour that can be interpreted as a descriptive norm. In the model of Brock & Durlauf [158], a choice between two competing scientific theories (*x* = 0) or (*x* = 1) is influenced by existing evidence but also by social factors captured by the mean choice $\bar{x}$ in the population. The utility function is:

3.8 $$u(x)=\underset{\mathrm{evidence-based}\;\mathrm{utility}}{\underbrace{A_{0}\pi (x)}}-\underset{\mathrm{conformity}}{\underbrace{A_{3}{\left(x-\bar{x}\right)}^{2}}}.$$

The authors showed that social interactions can lead a community consensus away from that theory which is superior by scientific criteria (i.e. the one that has the highest value of *π*).

With two actions, norm following (*x* = 1) and norm-breaking (*x* = 0), López-Pérez [102] defined the utility function as:

3.9 $$u(x)=\underset{\mathrm{material}\;\mathrm{pay-off}}{\underbrace{A_{0}\pi (x)}}-\underset{\mathrm{cost}\;\mathrm{of}\;\mathrm{norm}\text{-}\mathrm{breaking}}{\underbrace{A_{3}\bar{x}(1-x).\;}}$$

He used his model to offer a norm-based explanation for why many subjects in experimental games cooperate contrary to their material interest, cooperate in a reciprocal manner, and are willing to punish those who behave unkindly.

Azar [159] modelled tipping. Let *x* be the tip in percentage of the bill and $\bar{x}$ the average tip in the previous period. The value of $\bar{x}$ is viewed as a descriptive norm. Then the utility function is:

3.10 $$u(x)=\underset{\mathrm{material}\;\mathrm{pay-off}}{\underbrace{-cx}}+\underset{\mathrm{moral}\;\mathrm{satisfaction}}{\underbrace{yx}}-\underset{\mathrm{social}\;\mathrm{disapproval}}{\underbrace{A_{3}{\left(x-\bar{x}\right)}^{2},}}$$

where *c* is the bill size and *y* the strength of internalization of the tipping norm. The term *yx* captures the positive feelings obtained from tipping. Azar shows that if there are consumers with *y* > 0 who get moral satisfaction from tipping, tipping norms could stabilize (or even grow infinitely under specific extreme parameter conditions).

Azar [160] modelled workplace norms such as the refereeing time in an economics journal. Let *y* be the reviewer’s personally preferred time given their personal characteristic, how busy they are, their interest in the paper, etc. The utility function is

3.11a $$u(x)=-\underset{\mathrm{cost}\;\mathrm{of}\;\mathrm{deviating}\;\mathrm{from}\;y}{\underbrace{A_{1}{\left(x-y\right)}^{2}}}-\underset{\mathrm{conformity}\;\mathrm{with}\;\mathrm{existing}\;\mathrm{norm}}{\underbrace{A_{2}{\left(x-\bar{x}\right)}^{2},}}$$

where $\tilde{x}$ is the an existing (descriptive) norm. Azar postulated that the norm is given by a weighted average of the norm in the previous period and the average refereeing delay in the previous period:

3.11b $${\tilde{x}}_{t}={\tilde{x}}_{t-1}+\underset{\mathrm{learning}\;\mathrm{from}\;\mathrm{observations}}{\underbrace{\alpha ({\bar{x}}_{t-1}-{\tilde{x}}_{t-1}),}}$$

where *α* measures the weight of observations in the norm dynamics. Azar showed that the norm that gets established can be larger or smaller than the average preference $\bar{y}$ of individuals depending on the heterogeneity in the population. te Velde [161] modelled the effects of social image motivations on decision-making when the population is divided as to what is right. There are two possible meanings of social image: people may signal their adherence to their personal norm, or they may wish for others to approve their choices. Individuals differ in actions *x* and personal norms *y* and the utility function is:

3.12 $$u(x)=\underset{\mathrm{material}\;\mathrm{pay-off}}{\underbrace{A_{0}\pi (x)}}-\underset{\mathrm{cognitive}\;\mathrm{dissonance}}{\underbrace{A_{1}{\left(x-y\right)}^{2}}}+\underset{\mathrm{social}\;\mathrm{image}\;\mathrm{utility}}{\underbrace{A_{2}F(x,\bar{y}),}}$$

where the social image term *F* depends on the action chosen *x* and the distribution of types in the population. te Velde shows how distinct motives for maintaining social image lead to different outcomes in terms of consensus, hypocrisy, compromise, polarization and destructive posturing. Besides, using social incentives to change behaviour may easily backfire if heterogeneous norms, or approval and respect, are conflated. Earlier Brekke *et al.* [162] studied a similar model but without the last term in equation (3.12).

Houle *et al.* [163] studied cooperation and conflict in a society with multiple factions engaged in economic and political interactions. The model considers two interrelated games: an ‘economic game’, in which agents of identity-based factions and with different political power can cooperate (*x* = 1) or not (*x* = 0) in the production of a resource, and a ‘political game’, in which individuals devote a fixed proportion of their resources to a competition the results of which establish the rules of the economic game. The utility function is:

3.13 $$u(x)=-\underset{\mathrm{material}\;\mathrm{benefit}}{\underbrace{A_{0}\pi (x)}}+\underset{\mathrm{conformity}\;\mathrm{with}\;\mathrm{peers}}{\underbrace{A_{3}(2\bar{x}-1)x}}+\underset{\mathrm{conformity}\;\mathrm{with}\;\mathrm{state}}{\underbrace{A_{4}x_{s}x,}}$$

where *x*_*s*_ is the action of the most powerful faction (the state). Houle *et al.* showed that high conformity with the state (large *A*_1_) will stabilize cooperation, while high conformity with peers (large *A*_2_), can counter-intuitively, destabilize cooperation, because once a majority of low-power factions are defecting, the other factions are ‘pulled’ to defect as well. Houle *et al.* tested various modelling predictions using social unrest as a proxy for the breakdown of cooperation in society and data covering 75 countries worldwide between 1991 and 2016.

Yang *et al.* [164] used a game-theoretic model to explore the socio-cultural factors influencing mask-wearing during the COVID-19 pandemic. The utility of mask-wearing depended on perceived infection risk, strength of the descriptive social norm, institutional signals promoting mask-wearing, and individual sensitivity to these signals. The mask-wearing benefit correlated with the susceptible-exposed-infectious-recovered-susceptible infection model’s frequency of infected people. They found that increased pathogen spread or stricter policies could trigger a behavioural cascade, leading to full mask adoption. While cultural tightness can slow initial adoption (because people are more reluctant to modify their behaviour), it accelerates adoption once a tipping point is reached, helping establish mask-wearing as a norm. The tighter the culture, the more likely it is that collective mask-wearing will continue, even when the risk of infection decreases and policies are relaxed.

### (e) Dynamics of descriptive and personal norms

An important limitation of most models considered above is that they assume attitudes *y* remain constant. Next we discuss models explicitly accounting for the dynamics of attitudes.

Kuran & Sandholm [165] introduced a model of ‘cultural integration’, in which individuals have personal norms *y*, potentially related to their social identity, regarding behavioural acts *x*. However, they also benefit from coordinating their actions with others. We can capture these assumptions by an utility function:

3.14a $$u(x)=-\underset{\mathrm{material}\;\mathrm{pay-off}}{\underbrace{A_{0}{\left(x-\bar{x}\right)}^{2}}}-\underset{\mathrm{cognitive}\;\mathrm{dissonance}}{\underbrace{A_{1}{\left(x-y\right)}^{2}.}}$$

With constant personal norms, Kuran & Sandholm [165] show that the equilibrium behaviours of individuals reflect compromises between their own preferences and the need to coordinate with others. Kuran & Sandholm [165] also studied the case when personal norms change, by adapting the DeGroot model [107] of opinion change:

3.14b $$\frac{dy}{dt}=\underset{\mathrm{cognitive}\;\mathrm{dissonance}}{\underbrace{\alpha (x_{i}^{*}-y).}}$$

That is, each agent’s personal preference changes over time towards his current ‘action’ to reduce cognitive dissonance. In this case, preferences (*y*) and actions (*x*) converge to the initial mean preference ${\bar{y}}_{0}$. This convergence can be interpreted as the emergence of a single ‘melting pot’ scenario. Kuran & Sandholm [165] have extended the model to two partially segregated communities, where members have limited interactions with members from the other community. Their analysis focused on the extent of cultural segregation and the efficiency of policies aimed at preserving cultural distinctness or promoting cultural integration. Della Lena & Dindo [166] study different generalizations of the Kuran and Sandholm model.

Martins [167] considered a model in which individuals have a discrete set of alternative actions. Individual attitude/preferemce is specified by a probability distribution defined over this set. Each individual chooses the action with highest value, which means the utility function coincides with the personal norm. After choosing an action and observing groupmates’ behaviour, individuals update their personal norm using the Bayes rule. Numerical simulations on a network demonstrated the emergence of extreme personal norms, where individuals believe that one alternative is significantly superior to all others. Clusters consisting of individuals with similar attitudes arose, with central nodes in these clusters representing individuals with extreme personal norms.

Acharya *et al.* [168] considered strategic interactions between two agents. Utility function accounted for cognitive dissonance, conformity and a loss of utility owing to the deviation of the current personal norm from its initial value. They showed that at the equilibrium, personal norms match actions and that stronger conformity leads to large deviations from initial personal norms. Their results highlight that interactions between individuals expressing diverse perspectives can facilitate empathetic changes in actions.

Calabuig *et al.* [169–171] studied the coevolution of actions and personal norms in a linear public goods game with quadratic costs in heterogeneous groups. Both actions (*x*) and personal norms (*y*) are continuous variables. Individuals differ in the efficiencies of their efforts *s* and the shares *v* of the reward they secure from the good produced. These differences lead to differences in the efforts *θ* = *vs* maximizing individual material pay-off. Individuals are motivated by material pay-offs but also prefer to follow their personal norms *y*. The utility function is:

3.15a $$u(x)=\underset{\mathrm{material}\;\mathrm{pay-off}}{\underbrace{A_{0}\pi }}-\underset{\mathrm{cognitive}\;\mathrm{dissonance}}{\underbrace{A_{1}{\left(x-y\right)}^{2}.}}$$

After choosing an action maximizing utility and observing groupmates’ choices, individuals update their personal norms, driven by cognitive dissonance and conformity with groupmates. It is described by a DeGroot-type recurrence equation analogous to equation (3.14*b*) above:

3.15b $$y^{\prime }=y+\underset{\mathrm{cognitive}\;\mathrm{dissonance}}{\underbrace{\alpha (x-y)}}+\underset{\mathrm{conformity}}{\underbrace{\beta (\bar{x}-y)}},$$

where $\bar{x}$ is the average action and *α* and *β* measure the weight of the corresponding factors. The model allows for variation in all parameters.

**Figure 3.**
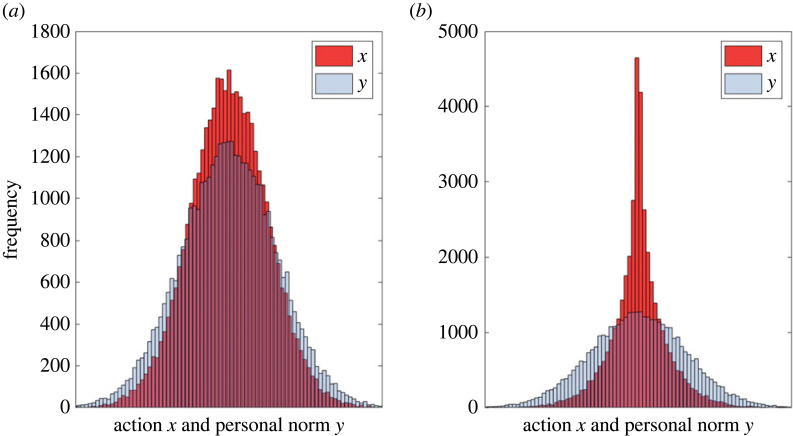
An example of equilibrium distributions of actions and personal norms in the model of Calabuig *et al.* [171] without (*a*) and with (*b*) evolution in personal norms. All averages are close to $\bar{\theta }$ but the variance in *y* is larger than that in *x* on the left but smaller on the right. The distribution of *θ* and the initial distribution of *y* are normal with same mean and standard deviations. The distributions of *A*_*i*_, *α*_*i*_, *β*_*i*_ are uniform on [0, 1]. The population size is 40 000. (Online version in colour.)

Calabuig *et al.* [169] show that the population evolves to an equilibrium with the average action $\bar{x}$ and the average personal norm $\bar{y}$ matching $\bar{\theta }$. At the same time, individuals deviate from the values *θ* maximizing their pay-offs. At equilibrium, the observed variances satisfy the inequalities: var(*y*) ≤ var(*x*) ≤ var(*θ*). Their results predict that (cultural) variation in personal norms and behaviour increases with the variance of skills (var(*s*)), the average group skill level $\bar{s}$ and the variance of the income sharing rule (var(*v*)). Increasing conformity (i.e. larger *β*) decreases this variation while increasing cognitive dissonance (i.e. larger *α*) or the weight of material factors (larger *A*_0_) have opposite effects. Figure 3 illustrates that ignoring the fact that personal norms can change can lead to very different predictions about the equilibrium distributions of actions and beliefs.

Calabuig *et al.* [171] used the above model to study the effects of culture on group productivity. They demonstrated that individualism increases the equilibrium efforts of individuals with above-average revenue and decreases them for those with lower revenues. Conversely, collectivism raises the equilibrium effort of individuals with below-average revenue and reduces it for high earners. In teams with diverse skills, individualism can affect both team revenue and costs depending on specific team parameters. In homogeneous teams, individualism only increases costs, but with unequal revenue sharing, full collectivism maximizes team production. The optimal balance between individualism and collectivism depends on the team’s income distribution and skill diversity. Lastly, the team’s culture can either amplify or mitigate the changes in skill or income distribution within the team.

Building on earlier work [172], Zino *et al.* [173] considered two possible actions (*x* = ±1), with the attitude *y* which can take any value within the range [−1, 1]. Individuals update their actions and opinions after interacting on a two-layer network. The utility function includes the terms for cognitive dissonance and for material pay-offs from coordination with neighbours in the so-called ‘influence layer’. Attitudes are updated according to a DeGroot-type model weighting communications and observations on the ‘communication layer’. The model exhibits a range of dynamics: rapid shifts to new sets of beliefs, where the majority adopts an innovation, or development and maintenance of an unpopular norm, where despite overwhelming support for an innovation, individuals fail to embrace it. Under some conditions the community favours the status quo over any innovation.

Mo & Sun [174] extend the above model by introducing an ‘opinion regulator’, an agent who can communicate with some nodes/individuals affecting their opinions and ‘impulsive stimulation’, a periodic reward or punishment for a specific behaviour administered to some individuals to promote or inhibit choosing this behaviour. Mo & Sun discuss optimal strategies of opinion regulating and impulsive stimulation for shifting behaviours in the population.

Aghbolagh *et al.* [175] used a similar model but with additional utility function components describing individual prejudices (unchangeable personal norms) and an external influence source. They identify the conditions necessary for the emergence and stability of polarized equilibria, in which the population divides into two factions endorsing and pursuing different courses of action. They also study conditions for pluralistic ignorance, when a social group mistakenly infers the opinions of others based on observed actions.

### (f) Dynamics of actions and descriptive, personal and injunctive norms

We are aware of only one paper jointly modelling the dynamics of normative ($\tilde{y}$) and empirical ($\tilde{x}$) expectations in addition to actions (*x*) and personal (*y*) norms [176]. Inspired by recent behavioural experiments [119,123,126–128,132], Gavrilets [176] described quantitatively the dynamics of these variables in social dilemmas. Besides social influences by peers, Gavrilets’ model also accounted for the influence by an external authority promoting a particular action *G*. Each individual chooses an action *x* to maximize the subjective utility function

3.16a $$\begin{array}{cc} u(x) & =\underset{\mathrm{material}\;\mathrm{pay-off}}{\underbrace{A_{0}\;\pi (x,\tilde{x})}}-\underset{\mathrm{cognitive}\;\mathrm{dissonance}}{\underbrace{\frac{1}{2}\;A_{1}{\left(x-y\right)}^{2}}}-\underset{\mathrm{disapproval}\;\mathrm{by}\;\mathrm{peers}}{\underbrace{\frac{1}{2}\;A_{2}{\left(x-\tilde{y}\right)}^{2}}}\\ & -\underset{\mathrm{conformity}\;w/\mathrm{peers}}{\underbrace{\frac{1}{2}\;A_{3}{\left(x-\tilde{x}\right)}^{2}}}-\underset{\mathrm{compliance}\;w/\mathrm{authority}}{\underbrace{\frac{1}{2}\;A_{4}{\left(x-G\right)}^{2}.}} \end{array}\}$$

After taking actions and observing groupmates’ behaviour, the attitude and beliefs of the individual change as described by the linear deGroot-type recurrence equations:

3.16b $$y^{\prime }=y+\underset{\mathrm{cognitive}\;\mathrm{dissonance}}{\underbrace{\alpha_{1}(x-y)}}+\underset{\mathrm{conformity}\;w/\mathrm{peers}}{\underbrace{\beta_{1}(X-y)}}+\underset{\mathrm{compliance}\;w/\mathrm{authority}}{\underbrace{\gamma_{1}(G-y),}}$$

3.16c $${\tilde{y}}^{\prime }=\tilde{y}+\underset{\mathrm{social}\;\mathrm{projection}}{\underbrace{\alpha_{2}(y-\tilde{y})}}+\underset{\mathrm{learning}\;\mathrm{about}\;\mathrm{others}}{\underbrace{\beta_{2}(X-\tilde{y}),\;}}+\underset{\mathrm{compliance}\;w/\mathrm{authority}}{\underbrace{\gamma_{2}(G-\tilde{y}),}}$$

3.16d $$\begin{array}{rl} \mathrm{and}\;{\tilde{x}}^{\prime } & =\;\tilde{x}+\underset{\mathrm{logic}\;\mathrm{constraints}}{\underbrace{\alpha_{3}(\tilde{y}-\tilde{x})}}+\underset{\mathrm{learning}\;\mathrm{about}\;\mathrm{others}}{\underbrace{\beta_{3}(X-\tilde{x}),}}\\ & \;+\underset{\mathrm{compliance}\;w/\mathrm{authority}}{\underbrace{\gamma_{3}(G-\tilde{x}),}} \end{array}$$

where prime indicates the next time step, *X* is the average action of groupmates observed by the focal individual (so different individuals can have different *X*), and *α*_*i*_, *β*_*i*_, *γ*_*i*_ are non-negative constant coefficients measuring the strength of the corresponding forces.

**Figure 4.**
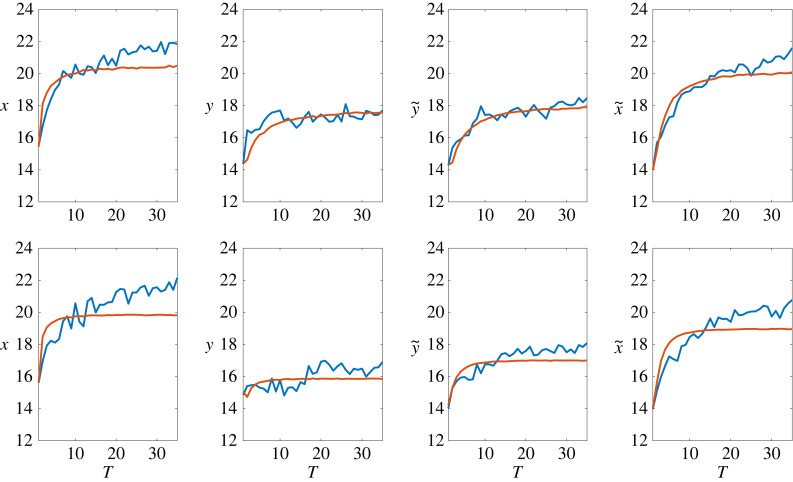
Observed (blue) and simulated (red) mean trajectories. The ‘simulated trajectories’ were obtained by repeatedly iterating dynamic equations describing the model using the obtained estimates of parameters and the actual individual data in the first round. Shown are at top: the results with no messaging; shown are at bottom: the results with messaging (after fig. S6 in [177].) (Online version in colour.)

Gavrilets [176] examined social interactions characterized by quadratic pay-off functions. With no messaging and in the absence of ‘stubborn’ individuals who refuse to change, the population progresses towards a state where the average behaviour aligns with behaviour maximizing individual material pay-offs, in agreement with standard game-theoretic models. On average, individuals develop attitudes and beliefs justifying (or matching) their behaviours. In equilibrium, substantial inter-individual variability exists in all variables, reflecting individual psychological traits. With messaging by an external authority, long-term equilibrium encapsulates a balance of diverse forces, often deviating from game-theoretic predictions. Attempts by an external authority to direct group behaviour can trigger an opposing behaviour (backfiring effect). Gavrilets [176] also studied how various factors can affect differences in tightness/looseness of social norms between groups and societies, highlighting societal heterogeneity, societal threats, authority effects, cultural variations in collectivism versus individualism, population size and subsistence style as significant factors. Tverskoi *et al.* [177] tested this model using data from a long-term common pool resources experiment without and with messaging promoting group beneficial actions. Figure 4 shows that the match between model-based predictions and observed data is good.

Tverskoi *et al.* [178] adopted the model developed in [176] to investigate new technology diffusion using a model encompassing individual proficiency in the technology, shifts in attitudes (*y*) and adoption decisions (*x*). The model predicts that early adopters exhibit low dissonance and peer conformity, but are swayed by authority. Also, individualistic societies fare better in early technology adoption, societies with strong normative factors and conformity with authorities promoting new technology achieve high adoption rates, those with high cognitive dissonance resist new technologies, and future-oriented cultures embrace innovations. The dynamic nature of personal norms is crucial for these predictions.

Gavrilets & Richerson [179] simplified the model in [176] to analyse evolution of food sharing in small-scale societies, involvement in political protests, and the impact of priming social identity in behavioural experiments. For each application, their approach provides different (or simpler) explanations of human behaviour compared to other methods. Moreover, they precisely determined and characterized the extent of discrepancy between individual actions and attitudes.

### (g) Evolutionary emergence of norm-utility

The various norm-utility models discussed above presume specific non-material utility function components. The emergence of norm-utility has been explored in several studies. Alger & Weibull [180] demonstrated that assortative interaction based on chosen strategies can lead to the evolution of individual utility functions, turning socially optimal behaviour into personal norms. Gavrilets & Richerson [181] explored the genetic evolution of the capacity to internalize social norms within populations undertaking collective actions. This model was expanded by Lozano *et al.* [182] for competitive within-group dynamics. Akcay & van Cleve [183] showed that populations engaging in social interactions could evolve to internalize the necessity to conform to majority behaviour. Kimbrough and co-workers [184,185] studied the origin of personal norms and normative expectations, accounting for individual differences in consumption utility. They found that injunctive norms could arise from minimizing overall consumption-related dissatisfaction as agents interact. If consumption utilities are unknown, personal norms could emerge from minimizing perceived dissatisfaction based on beliefs about others’ consumption utilities. Normative expectations emerge as individuals’ perceptions of others’ personal norms based on current information.

## 4. Discussion

By integrating norm-utility approaches with belief dynamics, recognizing cognitive forces, accounting for individual differences and considering the role of authority influences, we can effectively and flexibly model the emergence, persistence and evolution of social norms. Such models allow for a rich, multifaceted exploration of the complex coevolution of norms and beliefs over time and across different spheres of human life.

Several general patterns arise from the models discussed above. First, certain behavioural patterns can persist within populations for a long time. These could be some advantageous behaviours, like cooperation [102,103,163,176,179], but also behaviours detrimental for individuals’ material well-being or privately disapproved of [112,139,154–156]. Mechanisms contributing to the stability of such norms include preference falsification (publicly expressing preferences disagreeing with their true private ones [133]), pluralistic ignorance (mistakenly believing that one’s private beliefs are in the minority even if they are widely shared [9–11]), false enforcement (enforcing a norm privately disapproved of, [154]) or the ‘spirals of silence’ (hesitating to voice dissenting opinions or divergent behaviour [186]).

Psychological and cognitive processes play crucial roles in maintaining and transforming social norms. Among these processes, cognitive dissonance (and its consequences for behaviour and beliefs), having being widely modelled [112,135,138,154,161,165,168–171,173,175,176,178], stands out as a significant factor that can give rise to backfiring effects. Models show that imposing stricter penalties may surprisingly lead to an increase rather than a decrease in criminal behaviour [135]. Similarly, a heightened public shaming and disapproval of amoral conduct can unexpectedly contribute to an upsurge of such behaviour [138]. People’s reactions to messaging and nudging may steer them in the opposite direction of the intended one, and variations in social projection and cognitive constraints on beliefs can result in diverse dynamics of actions and preferences [176].

Models focusing on descriptive norms assume that people correctly identify them from observations, i.e. beliefs are correct [106,158,160,163,169–171,178]. When interactions happen on social networks, people have information only about the average behaviour among their social partners [175]. Only a few papers considered that people’s empirical expectations can differ from observed behaviours, and even less models look at the dynamics of injunctive norms [176]. Nevertheless, the models show that incorrect perception of norms will strongly affect group behaviour and belief dynamics. For example, one consequence are self-fulfilling prophecies—predictions that, by being made, directly or indirectly make themselves true [4]: if it is collectively believed that some behaviour is the norm, individuals are likely to conform to that behaviour, thereby making the prediction true [156,176].

Mathematical models depict dynamics leading to tipping points, where infrequent behaviour suddenly becomes widespread. This can happen after a significant shift in external circumstances (e.g. environmental or political) making a different behaviour more advantageous compared to previous practises. Alternatively, there may be a mass realization that long-held beliefs about personal circumstances, identity, or perceptions of others are flawed. More intriguingly, situations leading to tipping points can arise from minute changes. In mathematical models, this requires the existence of multiple equilibria such as those shown in figure 2. Alternative behaviours yielding higher pay-offs, strong conformity or mismatches between high pay-off strategies and authority-promoted norms promote multiple stable states. The exact conditions for tipping point dynamics largely depend on model specifics, parameters and belief distributions within the population.

Models predict that polarization in behaviour and beliefs can be sustained by differing behaviour pay-offs, allowing disparate belief systems to remain stable in the population despite varied societal advantages [112,161,162]. These models highlight how norms and beliefs are susceptible to manipulation by those with specific agendas. Models also suggest that norms and beliefs are highly susceptible to manipulation. Individuals or groups with particular agendas may exploit this vulnerability, significantly altering shared norms and collective beliefs. Individual and cultural differences can greatly impact social change dynamics and outcomes [164–166,169,171,176,178]. Additionally, the structure of social networks, including individual connections and information flow, significantly influences the spread of new behaviours and beliefs. Models also stress the importance of initial conditions, particularly the location of behaviour emergence, with some suggesting that innovations arising on a network’s periphery have a higher success rate.

Mathematical models of social norms dynamics provide an invaluable foundation for understanding how norms develop and evolve. Extending these approaches is crucial to more accurately reflect key factors shaping our societies. While cooperation and coordination have been successfully modelled using norm-utility approaches, other types of norms may require different methods. For example, the signalling norm [187] is described by a sequential game for which the norm-utility approach would not be practical to apply. Punishment of norm violators is pivotal for both the establishment and preservation of social norms [2,19,21,26–29]. Despite its importance, there has been relatively scant effort to integrate punishment mechanisms into norm-utility models [154,156,181]. Much of the modelling work on punishment has been conducted within the framework of evolutionary game theory, where individuals are generally pre-programmed to either penalize defectors or emulate those with the highest pay-offs [32,93,94]. Enhancing norm-utility models to more comprehensively include punishment mechanisms would substantially elevate both their realism and applicability. We also need to better incorporate network structure, the intricate web of relationships that influence the propagation of norms [62,188–190]. We need to address the emergence of social norms online, and the rise of new cultural authorities in digital spaces [191]. Also, we should account for the evolutionary emergence of differences in the parameters of utility functions and belief dynamics, considering how different cultural contexts shape individual and collective values, preferences and beliefs, and how these differences play out in social norm dynamics. Beyond network structure, intra- and inter-individual forces and culture, attention should be paid to how groups, their identities and between-group relationships are formed and change. In particular, the feedback loops between identity-linked social norms and forces changing the group boundaries may also be very important. Finally, we need more detailed and realistic models linked to tangible real-world processes, such as ecological and environmental shifts, economic fluctuations, or epidemiological trends. This would enrich our understanding of how rewards and penalties associated with different behaviours can shape the formation, persistence and change of social norms. Such enhanced models of social norm dynamics, if properly validated, parameterized and tested (e.g. [177]), could more accurately reflect the nuanced and complex reality of human social behaviour and be applied for mitigating various challenges faced by our society [192].

## Data Availability

This article does not contain any additional data.
